# EDUCATION AS A RISK FACTOR OF MILD COGNITIVE IMPAIRMENT—THE ROLE OF THE GUT MICROBIOME

**DOI:** 10.1093/geroni/igad104.0228

**Published:** 2023-12-21

**Authors:** Matthias Klee, Velma Aho, Patrick May, Rejko Krueger, Paul Wilmes, Anja Leist

**Affiliations:** University of Luxembourg, Esch-sur-Alzette, Luxembourg; Luxembourg Centre for Systems Biomedicine, Esch-Sur-Alzette, Luxembourg; Luxembourg Centre for Systems Biomedicine, Esch-Sur-Alzette, Luxembourg; Luxembourg Centre for Systems Biomedicine, Esch-Sur-Alzette, Luxembourg; Luxembourg Centre for Systems Biomedicine, Esch-Sur-Alzette, Luxembourg; University of Luxembourg, Esch-sur-Alzette, Luxembourg

## Abstract

**Background:**

Social determinants of health relate to an individual’s risk of MCI and dementia. However, pathways from identified modifiable risk factors such as education are not well understood, yet. While previous findings suggest distinct taxonomic signatures of the gut microbiome in dementia and MCI patients, and further education linked to its composition, we sought to test the possibly mediating role of the gut microbiome in the relationship between education and MCI. Method: Gut microbiome composition was ascertained with 16S rRNA gene amplicon sequencing. MCI classification was based on the Montreal Cognitive Assessment. Education in years was grouped (0-10, 10-16, 16+). Mediation analysis was conducted decomposing direct and indirect effects of education on MCI mediated by gut microbiome diversity (Chao1, Inverse Simpson, Shannon) or individual species at Genus level (ldm-med, permanova-med). Differential abundance analysis across education groups was conducted (ANCOM-BC, DESeq2). Result: After exclusion of participants with PD, below age 50, or with missing data, n=256 participants (n=58 with MCI) of the Luxembourg Parkinson’s Study were eligible for analysis (M[SD] Age=64.7[8.3] years). Education (16+ compared to 0-10 years of education) had a natural direct effect of NDE=0.36 (P<.01) on MCI, Chao1 included as mediator. We did not find significant mediation by gut microbiome composition or individual species.

**Conclusion:**

Our findings indicate direct effects of education not mediated by the gut microbiome. Taxonomic analysis suggests a signature linked to lower risk of dementia in higher educated individuals. Longitudinal research is needed to investigate associations over time.

